# Proposal for an electrostrictive logic device with the epitaxial oxide heterostructure

**DOI:** 10.1038/s41598-020-71631-5

**Published:** 2020-09-03

**Authors:** Md. Khirul Anam, Pratheek Gopalakrishnan, Ann Sebastian, Ethan C. Ahn

**Affiliations:** The Department of Electrical and Computer Engineering, The University of Texas at San Antonio, San Antonio, TX 78249 USA

**Keywords:** Engineering, Materials science, Nanoscience and technology, Physics

## Abstract

The possible use of electrostrictive materials for information processing devices has been widely discussed because it could allow low-power logic operation by overcoming the fundamental limit of subthreshold swing greater than 60 mV/decade in conventional MOSFETs. However, existing proposals for electrostrictive FET applications typically adopt approaches that are entirely theoretical and simulative, thus lacking practical insights into how an electrostrictive material can be best interfaced with a channel material. Here we propose an electrostrictive FET device, involving the epitaxial oxide heterostructure as an ideal material platform for maximum strain transfer. The ON/OFF switching occurs due to a stress-induced concentration change of oxygen vacancies in the memristive oxide channel layer. Based on finite-element simulations, we show that the application of a minimal gate voltage bias can induce stress in the channel layer as high as 10^8^ N/m^2^ owing to the epitaxial interface between the electrostrictive and memristive oxide layers. Conductive AFM experiments further support the feasibility of the proposed device by demonstrating the stress-induced conductivity modulation of a perovskite oxide thin film, SrTiO_3_, that is well known to serve as the substrate for epitaxial growth of other functional oxide layers.

## Introduction

The need to accelerate computing speed while maintaining the same or similar level of power consumption (i.e., enable a system-level benefit in an energy-delay product^1^) is one of the oldest and most challenging problems in microelectronics^2–4^. Although Moore’s law has driven the economics of the semiconductor industry over the past half century to enable an unprecedented improvement in microprocessor speed, the ever-increasing cost of computing power has still been a major issue in a wide variety of emerging applications. This is a direct consequence of the non-scalability of the subthreshold swing (SS) to below 60 mV/decade arising from Boltzmann statistics, which govern the operation of conventional MOSFETs (metal–oxide–semiconductor FETs). Electrostrictive FET^5^ is one of several steep subthreshold slope device concepts, including tunnel FETs^6,7^ and negative capacitance ferroelectric FETs^8,9^, that have emerged to achieve an SS value of less than 60 mV/decade. Electrostrictive FET works based on the principle of voltage-induced strain transduction by harnessing an electrostrictive (piezoelectric) material as a gate oxide layer that expands when exposed to an applied gate bias and consequently transduces an out-of-plane stress onto the adjacent channel material. Because this stress can change the electronic band structure of the semiconducting channel material (either bulk Si^5^ or 2D material^10^), the channel conductance could be modulated to provide the necessary ON/OFF switching for FET device operations. Owing to the internal feedback mechanism between electrostatic and electrostrictive potentials that gives rise to voltage amplification^10^, the sub-60 mV/decade subthreshold characteristic was predicted to appear in such an electrostrictive FET device structure.

In 2013, Hemert and co-workers have firstly reported a theoretical framework for the electrostrictive FET^5^. This work has attracted much attention by suggesting that strain modulation can open a new pathway to steep subthreshold slope devices. By contrast, state-of-the-art “strain device” technology at that time, which was based on the use of materials with built-in stress or lattice mismatch, resulted in a *constant* strain; it was not possible to *modulate* strain with external means (e.g., the gate voltage bias in a FET structure). A more scalable approach was proposed in 2016 by using a modern FinFET structure adopting a semiconducting two-dimensional (2D) material as an electrostrictive FET channel^10^. In addition to its capability to achieve the SS of sub-60 mV/decade, the 2D material’s ultra-thin body and electrostatic integrity^11^ offered great promise for the technology nodes beyond 10 nm. However, this work was also entirely simulative, and to date no one has experimentally demonstrated the electrostrictive FET device that is capable of logic operations. This is mainly due to the fact that a fundamental question of “how can an electrostrictive material be best interfaced with a channel material for maximum strain transfer?” still remains unanswered. A more recent work^12^ has experimentally demonstrated an electrostrictive FET with black phosphorus (BP)^13^ as a bandgap-engineered channel material. However, in this work the strain was applied through the substrate bending without the essential voltage-induced strain transduction process; therefore, this BP-based electrostrictive FET may find its best application in sensing (strain gauge) rather than computation. Fundamentally, the mobility values of these emerging 2D materials are never close to the state-of-the-art silicon and/or they still require much work to improve reliability^11^. In this context, the proposed device based on the epitaxial oxide heterostructure (growth techniques are well established to achieve the high-quality epitaxial interface^14,15^) is sharply distinguished from those of prior works and provides a practical route of constructing the electrostrictive FET for emerging logic device applications.

**Figure 1:**
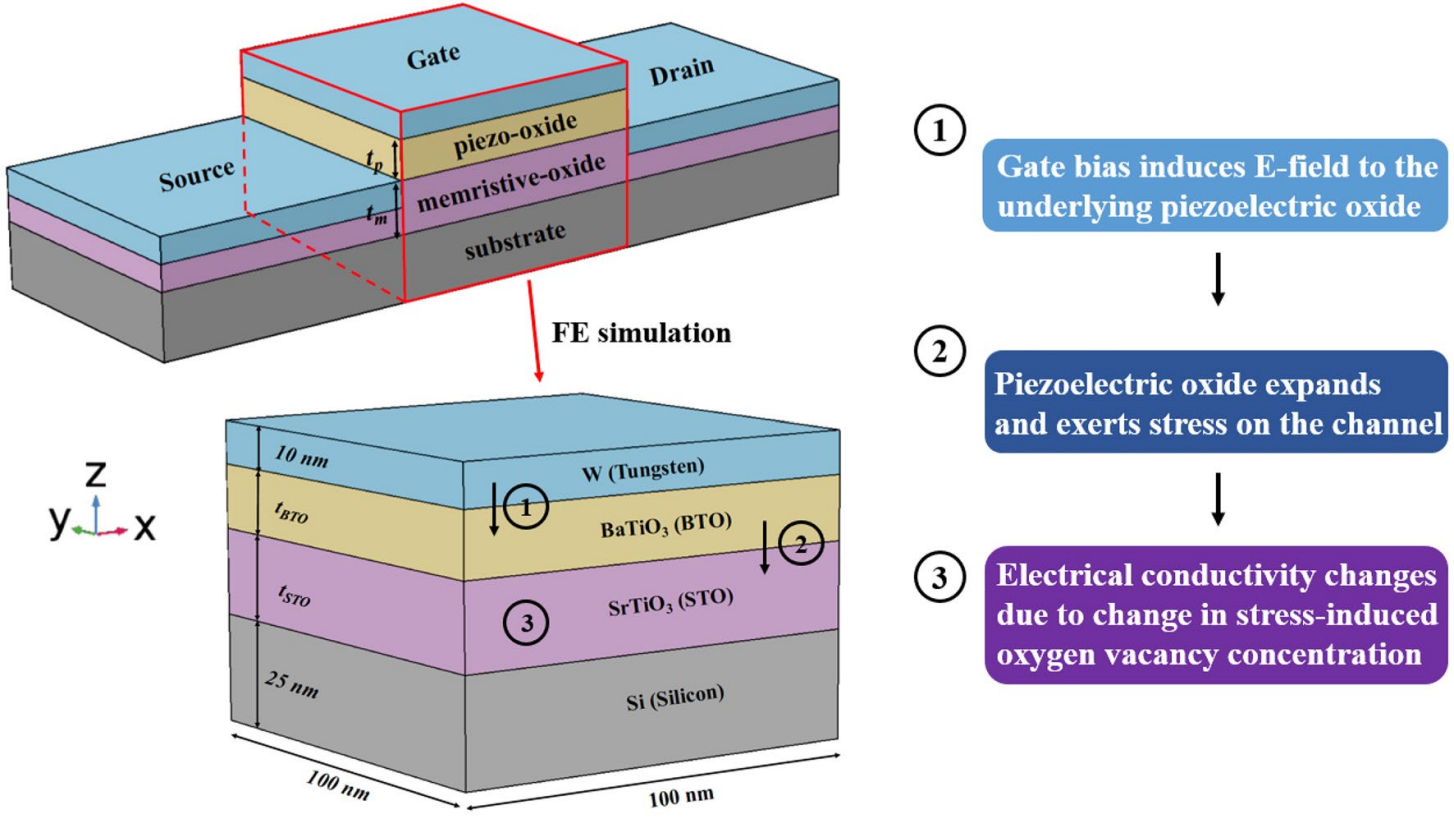
A 3-dimensional schematic of the proposed electrostrictive FET device structure. The FE (finite element) simulation is carried out on the heterogeneous oxide material stack highlighted in a red zone; W (tungsten) is used as the gate electrode contact, BTO and STO constitute the representative epitaxial oxide heterostructure, and Si (silicon) serves as the substrate. The thicknesses of the substrate and gate electrode layers are fixed at 25 nm and 10 nm, respectively, while those of piezoelectric and memristive oxide layers (t_p_ and t_m_) vary in the simulation to enable the scaling study. The flow chart on the right summarizes the operational principle of the proposed device structure.

The schematic diagram of the proposed electrostrictive FET device structure is shown in Fig. 1, where the perovskite oxide thin films of BaTiO_3_ (BTO) and SrTiO_3_ (STO) constitute an epitaxial oxide heterostructure. The epitaxial interface enables maximum strain transfer from an electrostrictive oxide thin film to a memristive oxide channel layer. The top oxide layer (BTO) serves as a piezoelectric gate oxide that provides an electric field-induced strain, while the adjacent bottom oxide layer (STO) is used as a memristive channel that alters its conductivity through modification of oxygen vacancy concentration by mechanical stress. By preserving the internal voltage amplification mechanism^10^ (will be revisited in the Discussion section), the proposed device structure will exhibit sub-60 mV/decade subthreshold conduction if experimentally developed. This will greatly help to reduce the supply voltage of integrated circuits. It is also important to note that the device channel layer is not made from a typical (or emerging) semiconducting material but, instead, is a memristive oxide. Using the memristive oxide makes it possible to induce a reversible change in conductivity of the electrostrictive FET channel while significantly increasing the operational range of temperature and bias voltage due to the much higher bandgap energy of oxide (E_g_ of single-crystalline STO is greater than 3.5 eV^16^). This article will serve as a quantitative feasibility study on the proposed device structure, enabling further studies (such as device fabrication and electrical characterization) on the development of electrostrictive logic.

## Methods

### Finite-element simulation

The COMSOL Multiphysics^17^ was used as a finite-element (FE) simulation tool to examine the strain transfer in the proposed epitaxial oxide heterostructure stack, including the gate terminal (tungsten) and the device substrate (silicon). The specific purpose of our simulative study is twofold: (1) estimating the stress (Pascal, N/m^2^) induced on the memristive oxide channel layer, given the minimal gate voltage bias applied (1 V was assumed throughout the simulation to represent the nominal supply voltage^18^), and (2) investigating the scaling trend of each functional oxide layer to provide useful insights into the device design and optimization. For the second purpose, we incorporated into the simulation a recent experimental finding for BTO thin films: the strong dependence of its piezoelectric coefficient (d_33_) on the BTO film thickness^19^.

The electromechanical behavior of the electrostrictive oxide layer (BTO)—namely, change in its shape (volume) under the application of an electric field, was modeled by a constitutive relation available in the COMSOL library. The coupling between electrical and mechanical effects is typically formulated in the FE simulation by either the strain-charge (inverse piezoelectric effect) or the stress-charge (direct piezoelectric effect) form. The strain-charge equation was used in this work (see Eq. 1 below), but it is worth noting that two constitutive relations can be easily derived from each other by the matrix transformation^20^. Indeed, we find that there is almost the negligible difference in simulation results between the two cases; the use of strain-charge physics generates slightly higher stress values than the stress-charge counterpart, but the overall trend remains the same. In the simulation, the substrate layer (silicon) was fixed and all other layers were allowed to experience volume expansion or contraction depending on the polarity of the gate voltage bias applied^21–23^. Electric potential was applied to the top surface of the BTO layer and its bottom surface was grounded to assume a full voltage drop across the piezoelectric layer (the simulated potential graph across the BTO layer is available in the Supplementary Information). The following two equations^20^ represent the fundamental electrostrictive physics incorporated in this simulation.

1 $$S = s_{E} T + d^{T} E$$

2 $$D = dT + \varepsilon_{0} \varepsilon_{r} E$$

*S*, *T*, *E*, and *D* represent strain (dimensionless, m/m), stress (Pascal), electric field (V/m), and electric displacement field (C/m^2^), respectively. $\varepsilon_{r}$ is the relative permittivity at constant stress, and $\varepsilon_{0}$ is the permittivity of the free space. The superscript *T* (in *d*^*T*^) denotes the transpose matrix operation. The specific material parameters in the compliance ($s_{E}$, 1/Pa) and coupling (*d*, C/N) matrices, full equations in the form of tensors, and other simulation details are available in the Supplementary Information.

### C-AFM experiments

We carefully designed and performed conductive atomic force microscope (C-AFM) experiments to apply localized mechanical pressure in the range of a few tens to hundreds of MPa (10^6^ N/m^2^) and simultaneously measure any change in conductivity (S/m) of the memristive oxide layer. A relatively thick (1 mm) single-crystalline STO film was used in this experiment to ensure that pressures as high as a few hundred MPa are well sustained (c.f., STO thicknesses of less than 30 nm were used in the simulation). Nevertheless, our experimental work with a separate STO sample piece successfully emulates the situation in the proposed device structure because the simulation results indicate that the maximum stress always occurs at the top surface of the STO layer and the use of STO films thicker than 30 nm yields a negligible difference (see the Results section for details).

## Results

**Figure 2:**
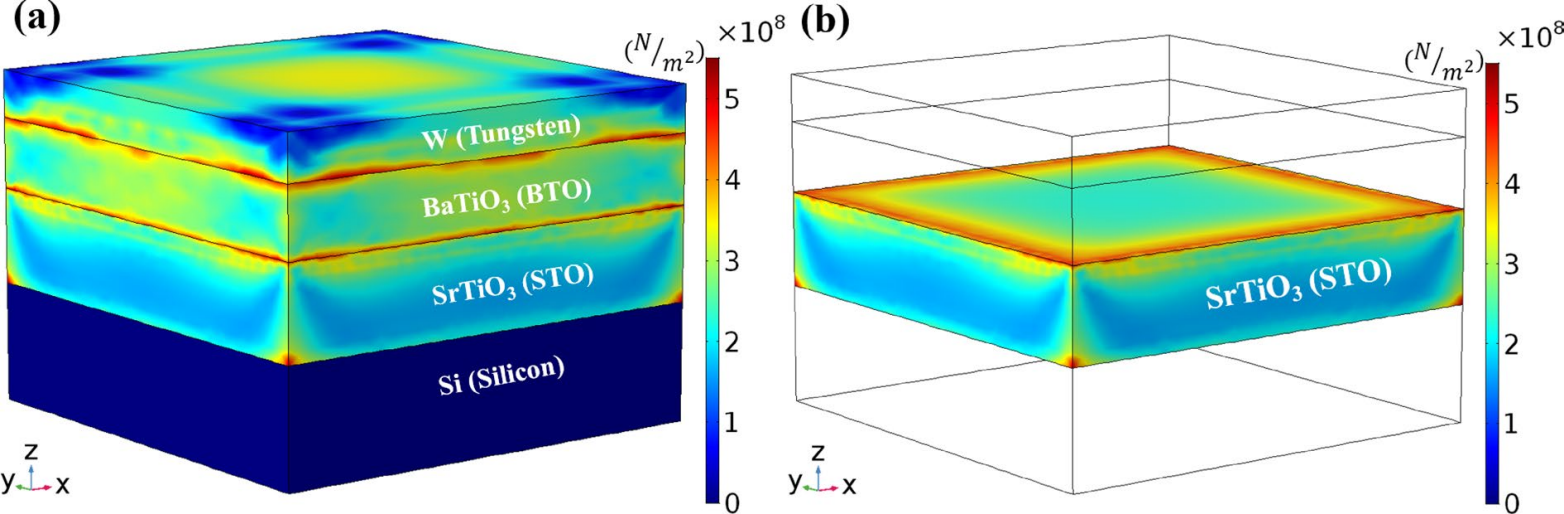
The electromechanical simulation results, showing the gate voltage-induced stress levels (Pascal, N/m^2^) for the whole epitaxial oxide heterostructure stack (**a**) and the STO channel layer only (**b**). Even with a small gate voltage bias applied (1 V as the nominal supply voltage), considerably high stresses of up to a few hundred MPa are induced owing to the sharp interface between the BTO and STO layers assumed in the simulation. This assumption has the strong experimental basis; the proposed structure is experimentally achievable by employing the epitaxial growth-capable PVD technique. In (**b**), it is clear that except for edges and corners where the known issue of stress singularity appears, the maximum stress occurs at the top surface of the STO layer along the z-axis.

Figure 2 shows the FE simulation results on the gate voltage-induced (von Mises) stresses for the whole epitaxial oxide heterostructure stack (including the contact and the substrate) of [W (10 nm)/BTO (15 nm)/STO (20 nm)/Si (25 nm)] (a) and the memristive oxide (STO) layer only (b). Under the application of a gate voltage bias of 1 V, the electrostrictive material (BTO) experiences realignment of its internal dipoles, giving rise to the crystal deformation (strain) along the z-axis. This electrical-to-mechanical energy conversion consequently induces out-of-plane stress onto the neighboring STO layer, as high as a few hundred MPa. It is important to note that the loss-free strain-to-stress transduction from BTO to STO will be only achievable if the interface quality is precisely controlled. Physical vapor deposition (PVD) techniques, capable of epitaxial growth, such as molecular beam epitaxy (MBE), can control the heterostructure interface at the sub-monolayer level, thus offering a viable experimental tool to implement the proposed device structure. It is also seen from the figure that geometric discontinuity of the simulated device structure, involving the heterogeneous thin film materials, causes stress singularity at corners and edges, which may be addressed by adaptive meshing or any other error handling techniques^24,25^. We focus on the stress induced at the center of the STO layer’s top surface, which always has the maximum value across its thickness (along the z-axis) (see Fig. 2b). The active channel region of the proposed electrostrictive FET device structure is comprised of this top surface of the memristive oxide layer, exposed to the maximum stress and thus, effective modulation of its conductivity.

**Figure 3:**
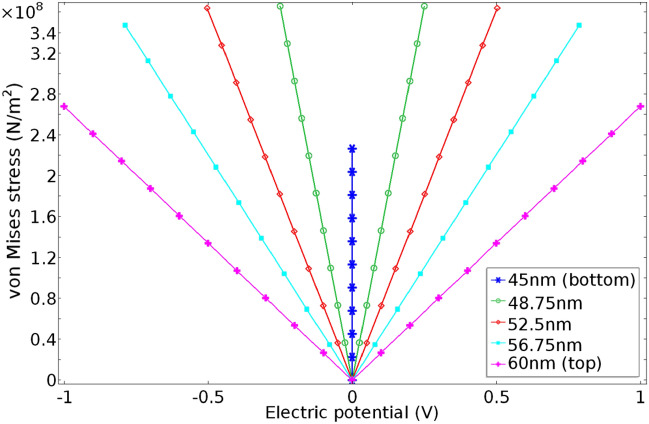
The phase diagram of the electrostrictive material (BTO) in the proposed electrostrictive device structure. For varying gate voltage biases (− 1 V to 1 V), both the induced stress and electric potential values are shown. By simulating five different cases of different coordinate values in the z-direction, it is found that higher stresses are induced at near the BTO bottom surface for the same electric potential value. Based on the presumed linear stress–strain relationship, the BTO bottom surface consequently produces the highest strain, which is transferred to the neighboring channel layer of the electrostrictive FET.

Figure 3 further displays a simulated phase diagram for the 15 nm-thick BTO layer under the gate voltage biases varying from − 1 V to 1 V; both the stress and electric potential values are shown for each position along the z-axis inside the layer (45 nm and 60 nm represent the bottom and top surfaces, respectively, and the other three z-coordinates are for the middle points in-between). The phase diagram provides a useful piece of information on the strain-to-stress transfer mechanism across the piezoelectric and memristive oxide layer interface. For example, it is found from the figure that for a given electrical potential, the stress due to electrostrictive deformation of the BTO layer is larger at positions closer to the BTO bottom surface (i.e., the BTO and STO interface). Consequently, the maximum stress on the adjacent STO layer occurs at the STO top surface near the BTO bottom surface.

**Figure 4:**
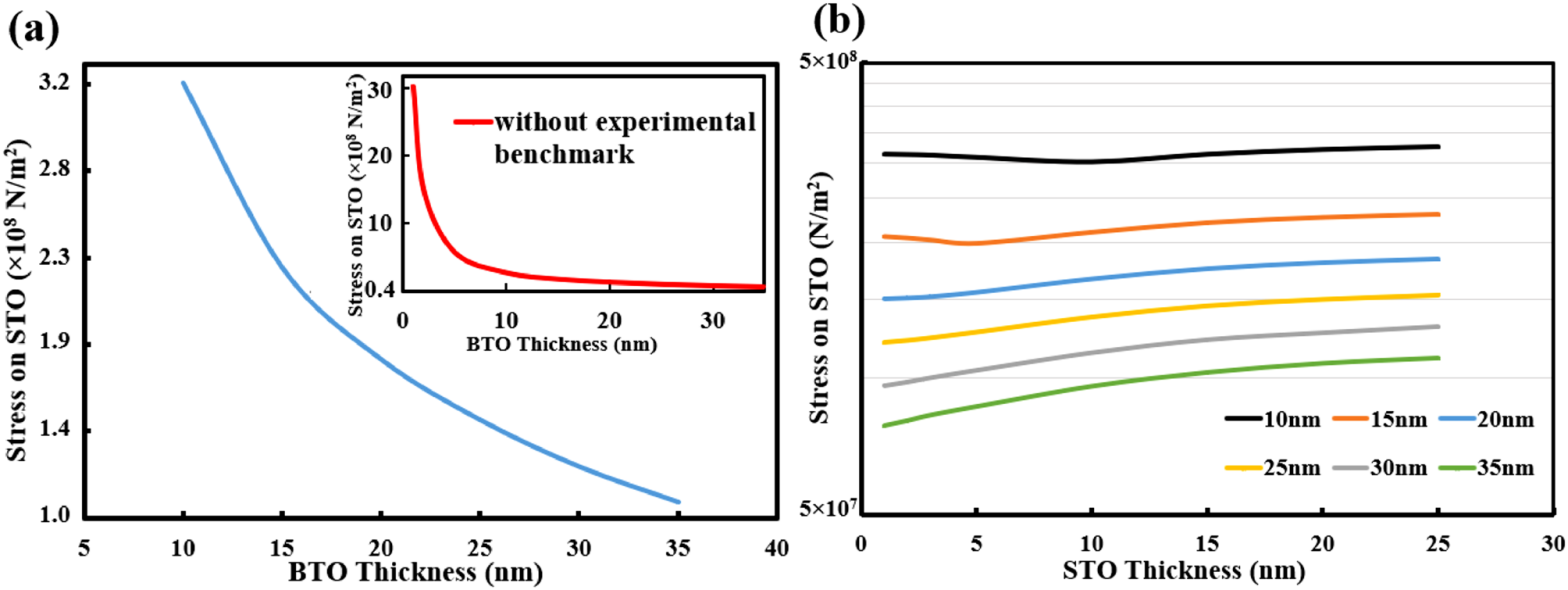
The thickness dependence of the maximum stress induced at the STO top surface for an applied gate voltage bias of 1 V. The impact of varying BTO thicknesses (**a**) or STO thicknesses (**b**) is shown. In (**a**), the experimentally measured electrostrictive coefficient values (d_33_) of BTO thin films were used, enabling us to incorporate the dependence of BTO’s piezoelectric strength on its thickness for the experimentally benchmarked simulation. The inset in (**a**) simulates the case of using the built-in material parameter values available in the COMSOL library, including the d_33_ value for a bulk BTO (d_33_ fixed for all different BTO thicknesses). In (**b**), the stress levels on STO films of varying thicknesses (2 nm to 25 nm) are shown for the BTO thicknesses ranging from 10 nm to 35 nm (legends). It is noted that for STO thicknesses larger than 30 nm, the stress tends to be almost independent of the STO thickness.

We next investigate in Fig. 4 the scaling behavior of the stress that is induced at the top surface of STO (i.e., how it changes with different thickness dimensions of the BTO and STO layers). It is highlighted that our simulative study features a practical scenario with a strong experimental basis. A very recent experimental study has reported that BTO thin films (epitaxially grown on (001) STO substrates) exhibit a considerable decrease in its electrostrictive coefficient value (d_33_) with decreasing film thicknesses^19^; d_33_ of 10 nm BTO was measured to be less than 2 (pm/V) while that of 80 nm BTO was 20.5 (pm/V). This experimental finding was directly incorporated into our simulation, making it possible to accurately predict the dependence of stress at the STO top surface on the BTO thickness. Figure 4a shows the result from the experimentally benchmarked model (i.e., d_33_ values vary for different BTO thicknesses) while the inset presents the case of using the fixed d_33_ value (8.56 × 10^–11^ (C/N) is available in the COMSOL library for a bulk BTO material) for all BTO thicknesses. Although the stress induced on STO (the STO thickness is fixed at 20 nm) increases with decreasing BTO thicknesses in both cases, the stress value is found to be slightly reduced in the experimentally benchmarked case. This is apparently due to inclusion of the piezoelectric coefficient values directly measured for BTO thin films, which were used for all other simulation results presented in this work. The graphs of stress vs. BTO thickness could also be plotted for different STO thicknesses; for a wide range of STO thicknesses (from 25 to 1 nm), similar observations were made. The impact of thicknesses of both the BTO and STO layers is clearly summarized in Fig. 4b, which simulates the stresses on the STO surface for different STO thicknesses (on the x-axis as the first independent variable) while varying the BTO thicknesses as well (on the figure legend as the second independent variable). It is observed that as compared with the BTO thickness, the STO thickness does not alter the results significantly. This could mean that requirements become less stringent for the STO layer thickness when designing the growth experiment for the BTO/STO epitaxial oxide heterostructure.

**Figure 5:**
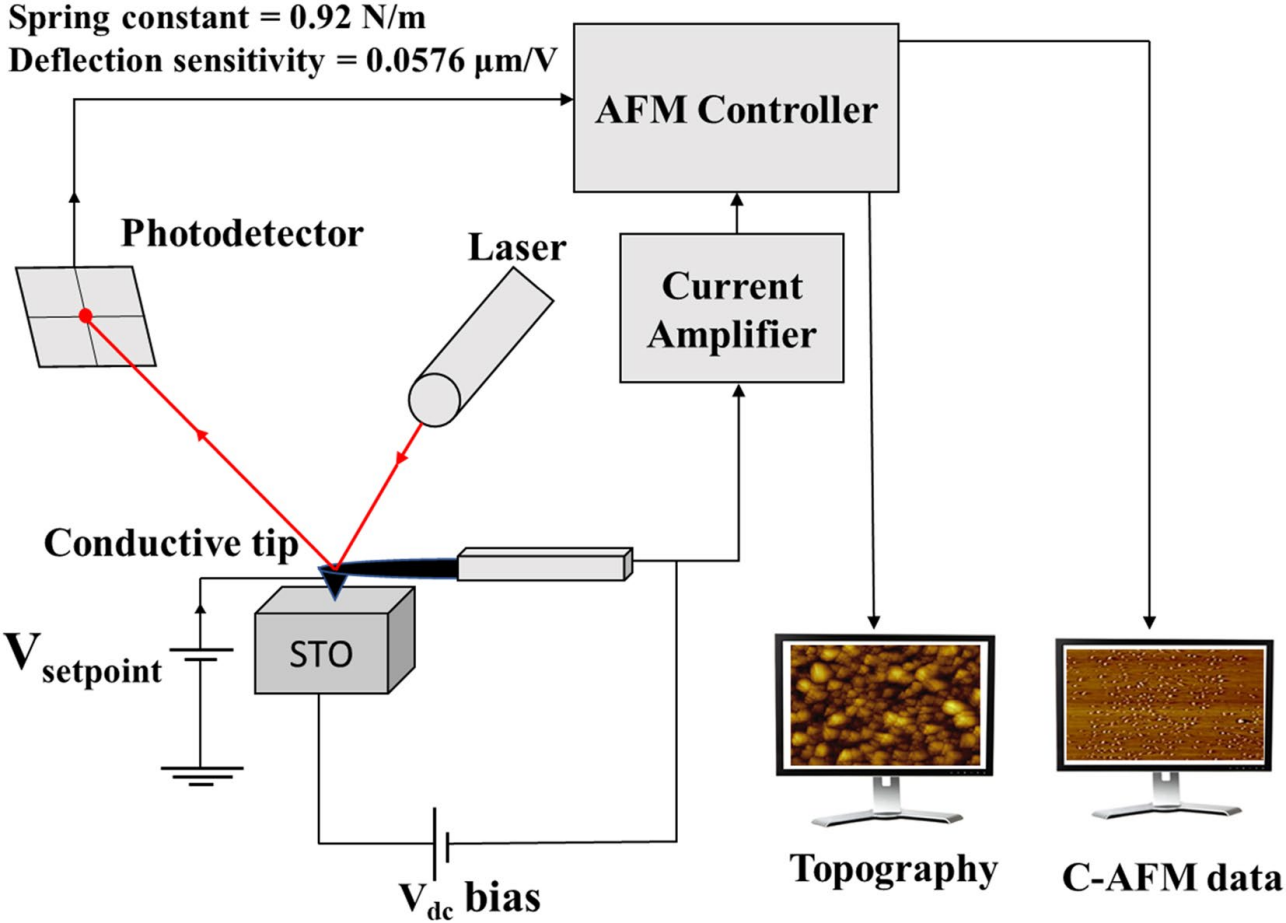
A schematic diagram of the conductive AFM (C-AFM) measurement setup. The DC voltage bias (V_dc_) makes it possible to measure the electrical responses upon the application of mechanical pressures while V_setpoint_ of varying values leads to different pressures applied onto the STO sample. Precise conversion from V_setpoint_ (in V) to mechanical pressure (in Pascal) was executed by calibrating the spring constant and the deflection sensitivity of the experimental setup and using the Hooke’s law.

Lastly, we present our C-AFM (Bruker Innova) experimental results on stress-induced conductivity modulation of STO. To ensure accurate measurements, we carefully designed and calibrated the experimental setup as shown in Fig. 5. A commercially available conductive tip (Asyelec.01-R2) was used to apply a bias voltage (V_dc_ ~ 2 V) while scanning through the top surface of the STO sample. This enables simultaneous measurements of electrical responses (current) during the experiment that are amplified and fed to the AFM controller. A force (pressure) was then applied to the STO sample surface with the C-AFM cantilever tip in the contact mode. Varying forces were applied by changing the set-point voltage (V_setpoint_, kept below 3 V to prevent any possible damage to the measurement system) of the setup; this makes the cantilever tip accommodate different heights and thus exert different pressures. Importantly, conversion from the control parameter (V_setpoint_) to the mechanical pressure (N/m^2^) was executed through precise calibration of the C-AFM measurement setup, yielding the spring constant of 0.92 (N/m) and the deflection sensitivity of 0.0576 (μm/V). Details of the experimental setup and calibration, and equations used, are available in the Supplementary Information.

**Figure 6:**
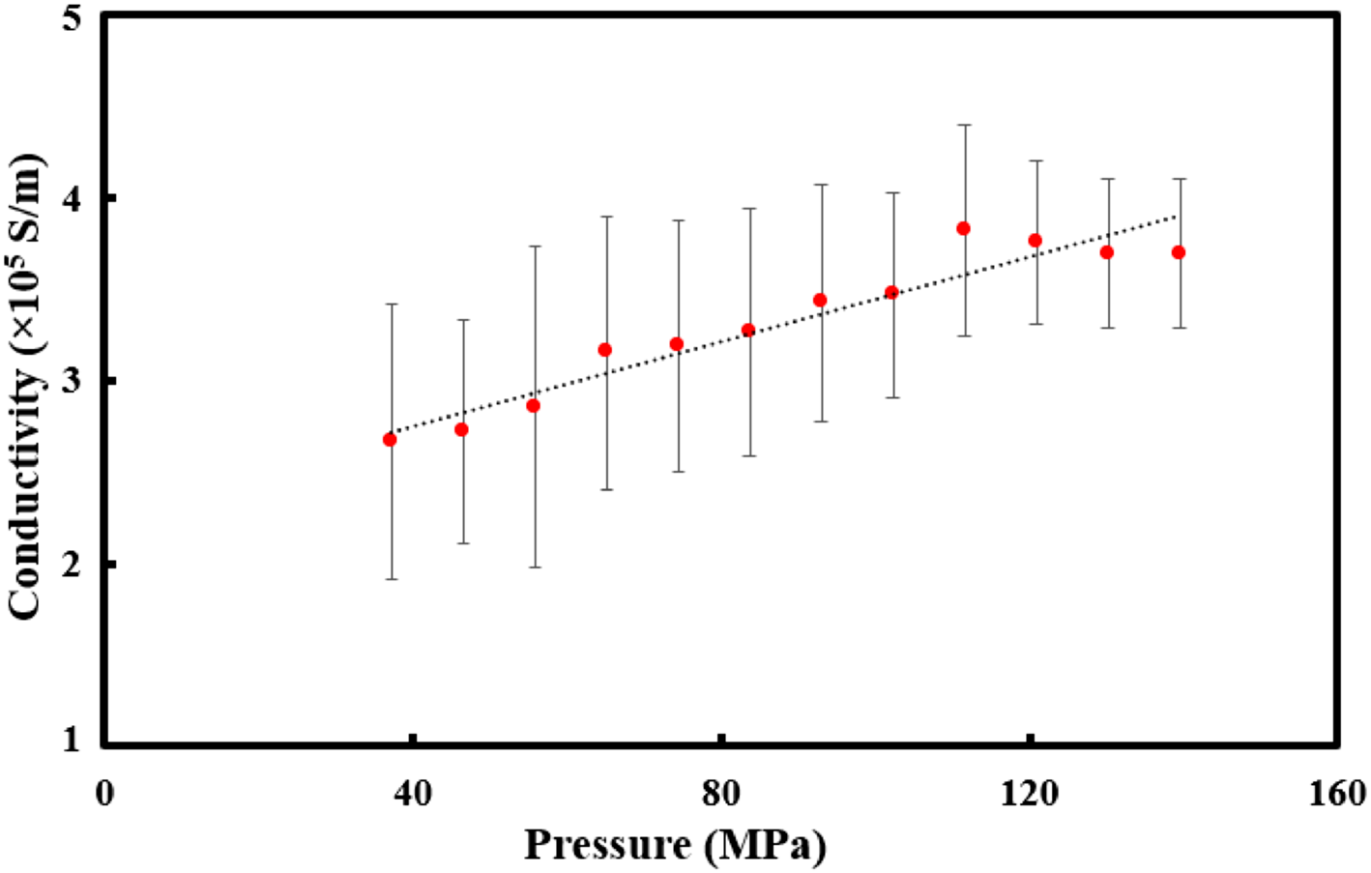
The conductivity of STO thin films under varying mechanical pressures applied in the C-AFM experimental setup. Each experiment was performed repetitively (10 times) and both the mean (data points) and standard deviation (error bars) values are presented in the figure. This ensures the statistically meaningful data. A clear trend of increase in electrical conductivity with increasing pressure is observed, which is best attributed to creation of oxygen vacancies due to the mechanical stress in STO.

Figure 6 shows the measured electrical conductivity (S/m) of the pristine (undoped) STO sample for applied pressures (MPa) varying from 40 to 140 MPa (corresponding to V_setpoint_ values varying from 0.8 V to 3 V in the C-AFM experimental setup in Fig. 5). To ensure statistically meaningful data, each experiment was performed repetitively (10 times) and both the mean (data points) and standard deviation (error bars) values were presented in the figure. A clear trend of increase in electrical conductivity with increasing pressure was observed, which is best attributed to creation of oxygen vacancies due to the mechanical stress in STO^26^. Controlling the electrical behavior of perovskite oxide thin films by the oxygen content has been possible due to a change in the crystalline structure (lattice parameter, tetragonality, etc.) with the varying amount of oxygen vacancies^27^. This is known to significantly affect the dielectric properties, leakage conduction mechanisms, and the resistive hysteresis of the oxide material. The considerably high electromechanical coupling sensitivity of ~ 1,400 Sm^-1^ MPa^−1^ was obtained from the linear fit, thus demonstrating that electrical transport characteristics of perovskite oxide thin films can be largely tuned by external stimuli such as mechanical pressure. According to our simulative studies presented in earlier sections, the stress is only induced in STO when the positive gate voltage bias is applied to the top contact of the epitaxial oxide heterostructure. Because our experimental results further support the idea that stress-induced conductivity modulation is reversible between high conductance (upon high pressure) and low conductance (upon low pressure) states, we argue that the proposed device is feasible and has the potential to serve as the next generation of sub-60 mV/decade logic devices. Fundamentally, it will spark various research initiatives in using the oxide thin films for emerging electronic applications (other than conventional CMOS components such as gate oxides or insulating trenches), including the electrostrictive FET channel.

## Discussion

From the experimental point of view, the proposed electrostrictive FET structure can be implemented by directly growing the epitaxial oxide heterostructure on the silicon substrate and then employing necessary lithographic patterning processes to define the contact electrodes. The advanced oxide-MBE technique can best render the oxide heterostructure interfaces as sharp as possible^28–30^, but other inexpensive, yet epitaxial growth-capable PVD techniques, such as pulsed-laser deposition (PLD), may also be revisited to optimize the growth recipe and parameter space for maximum strain transfer. It is noted that the proposed device structure (Fig. 1) has the STO layer fully extended to even below the source and drain contacts. Although only the STO layer’s top surface below the piezoelectric oxide layer participates in the device operation, this structure is much easier to fabricate than the case where STO only exists under the piezoelectric layer. This is because in the letter case, etching completely the STO layer outside the active device area can be nontrivial. The proposed structure can also help avoid any unnecessary device malfunctions because otherwise, the source-to-drain current may bypass the STO layer through the silicon substrate.

In addition to serving as the buffer layer for on-silicon epitaxial growth of other functional oxide thin films, STO enables the device functionality (i.e., ON/OFF switching) by its unique memristive characteristics (see the Supplementary Information for measured current–voltage hysteretic curves of the STO thin film). Similar memristive behaviors have already been reported for doped STO and other organic–inorganic hybrid perovskite oxide materials^31,32^. It has been well understood that (1) stress can facilitate spontaneous formation of oxygen vacancies inside a complex transition-metal oxide system by tuning the oxygen vacancy formation energy^33^, and (2) conductance of a memristive device is directly dictated by oxygen vacancy concentration inside the oxide thin film^34^. Therefore, demonstration of the stress-induced conductivity modulation for the memristive oxide layer (Fig. 6), which is the key experimental support for our proposal, should serve as the right experimental stage moving forward to novel device applications of the functional oxide thin film.

The operation of our device begins with the electrostrictive effect of the piezoelectric oxide material. As described in Eq. (3), the strain (*S*, defined as the piezo-oxide thickness ratio of Δt_p_ to t_p_) is resulted from the applied electric field (*E*) with a set of parameters (α, β, γ) that are specific to the choice of a piezoelectric oxide material. Next, the stress-induced conductivity modulation of the memristive oxide material demonstrated in this work (Fig. 6) suggests a strong correlation between the out-of-plane stress (*T*) and the defect (oxygen vacancy) formation energy. It has been demonstrated that in-plane tensile strain (accompanying out-of-plane compressive strain) lowers the oxygen vacancy formation energy of the perovskite CaMnO_3_^35^, thus facilitating the creation of oxygen vacancies and increasing the conductivity. Researchers may find it a very useful study to model the impact of out-of-plane stress to the oxygen vacancy formation energy for the memristive perovskite oxide such as STO. In our device structure, the strain (of the piezo-oxide) in Eq. (3) and the stress (of the memristive oxide) are coupled through Eq. (4), where *η* is the important parameter that quantifies the ratio of strain-to-stress transduction from the electrostrictive material to the channel material (i.e., *T* = *η*Δt_p_). It is noted that the sharp epitaxial interface between the piezoelectric and memristive oxide interface ensures that *η* is maximized, thus contributing to the improvement in subthreshold swing.

3 $$S = \frac{{\alpha E^{2} }}{{\beta + \gamma E^{1.5} }}$$

4 $$T = \eta t_{p} S$$

In a conventional MOSFET, the subthreshold slope (*SS*) is limited to 60 mV/decade because the electrostatic surface potential (Ψ_S_) cannot exceed the gate bias potential (Eq. 5). However, the additional potential component (Ψ_E_ = Ψ_E_(*η*)) appears in our device structure due to the electrostrictive creation of a high-conductivity conduction path, making the total channel potential always greater than Ψ_S_. This is referred to as an internal voltage amplification (i.e., $\frac{{\partial ({\Psi }_{S} + {\Psi }_{E} )}}{{\partial qV_{GS} }} > 1$), arising from the interplay between the electrostatic and electrostrictive potentials. This leads to the sub-60 mV/decade subthreshold conduction as already simulated in the previous work, assuming the ideal interface between the piezoelectric oxide and channel layers^10^.

5 $$SS = \left( {\frac{{\partial \log I_{DS} }}{{\partial V_{GS} }}} \right)^{ - 1} = \frac{{\frac{{k_{B} T}}{q}\ln 10\left( { = 60\frac{mV}{{decade}}} \right)}}{{\frac{{\partial (\Psi_{S} + \Psi_{E} )}}{{\partial qV_{GS} }}}}$$

The proposed electrostrictive FET structure, involving the epitaxial oxide heterostructure as the active device region, promises a rare combination of high-speed performance and low power consumption, along with excellent reliability and scalability features. The ON-state current density of greater than 10 mA/μm is achievable for the electrostrictive FET device with its memristive oxide channel of 10 nm in length and 10^5^ S/m in conductivity^10^. For the same device, the sub-60 mV/decade subthreshold slope is accompanied due to the positive feedback mechanism between electrostatic and electrostrictive potentials, already predicted^5^ and demonstrated^10^ for a general form of electrostrictive FETs. Moreover, the use of an oxide thin film as the FET channel will lead to negligible performance degradation even at a wide temperature or bias operating range due to the much higher bandgap energy of oxides (than typical semiconducting channels). Our scaling study also proves that the proposed device has the scaling advantage; the higher stress is resulted in when the thinner piezoelectric layer is used (Fig. 4a). Lastly, we highlight that energy-efficiency of the proposed device structure could be much improved with the use of other possibly stronger piezoelectric oxide layers. This is because the voltage required to induce the stress in the memristive oxide channel layer could be reduced. Thus, further development efforts for new piezoelectric materials that can be epitaxially grown on top of a memristive oxide layer may be requested to enable optimization of the electrostrictive device design and manufacturing.

## Summary

The combination of finite-element simulations and electromechanical coupling experiments provides a quantitative description of how an electrostrictive material can be best interfaced with a channel material for constructing a novel form of electrostrictive FET devices. Our proposal features the use of an epitaxial oxide heterostructure, involving the piezoelectric and the memristive oxide thin films as the strain-to-stress transducer and the FET channel, respectively. The feasibility of the proposed device structure was studied by demonstrating that considerably high pressures are induced on the memristive oxide channel surface with minimal gate voltage biases applied, and that its conductivity is well modulated by out-of-plane localized pressures. The experimentally benchmarked simulation model predicts the scaling behavior of each oxide layer; while the impact of the channel thickness is minimal, the use of a thinner piezoelectric layer can enhance the performance of the proposed electrostrictive FET device. The precisely calibrated C-AFM measurements lead to the observation of stress-induced conductivity modulation in the memristive oxide layer, which confirms our reasoning of using the memristive oxide as the FET channel. Advances in experimental techniques that support the development of new functional oxide materials (with enhanced piezoelectric performances) and the epitaxial growth of oxide heterostructures will pave the way to the next generation of logic devices with reduced power consumption and enhanced reliability features.

## Supplementary information

Supplementary information

## Data Availability

The authors declare that data supporting the findings of this study are available within the article.
