# Supplementary material for: Proposal for an electrostrictive logic device with the epitaxial oxide heterostructure
Source: Sci Rep. 2020 Sep 3;10:14636. doi: 10.1038/s41598-020-71631-5 (PMC7471687; doi:10.1038/s41598-020-71631-5)
Supplement: Supplementary file 1 — Supplementary information [file 41598_2020_71631_MOESM1_ESM.pdf]

[Supplementary Information]

Proposal for an Electrostrictive Logic Device with the Epitaxial Oxide Heterostructure

Md. Khirul Anam[1], Pratheek Gopalakrishnan[1], Ann Sebastian[1], and Ethan C. Ahn[1,]*

[1]The University of Texas at San Antonio, The Department of Electrical and Computer Engineering, San Antonio, TX 78249, USA

*email: **ethan.ahn@utsa.edu**

Potential Distribution in the Strain-Transducer Layer

The gate voltage bias of 1V was assumed throughout the simulation. An ideal scenario with a full voltage drop across the BTO layer is emulated in such a way that the highest stress level is calculated at the memristive channel oxide layer (STO). **Fig. S1** shows the simulated electrical potential distribution inside the BTO layer along the z-axis. Alongside the phase diagram of BTO presented in the main manuscript (Fig. 3), this helps understand the strain transduction mechanism induced by the applied voltage bias.

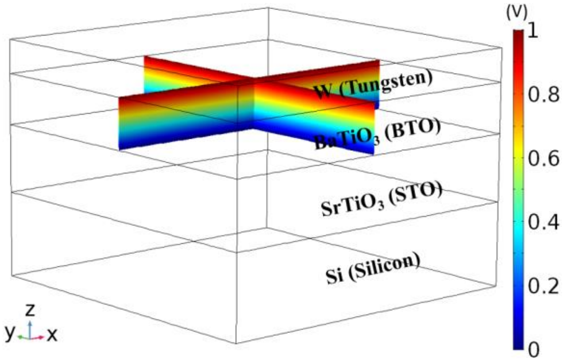

Figure S1. An electrical potential distribution throughout the piezoelectric material layer under an applied gate voltage bias of 1V.

Tensor Equations, Material Parameters, and Other Simulation Details

Fundamental electrostrictive physics utilizes a compliance matrix, s_E (the tensor rank of 4), a coupling matrix, d_{ET} (the tensor rank of 3), and the relative permittivity (ε_{rT}) at constant stress (the tensor rank of 2) [1]. Full tensor equations that correspond to Eq. (1) and (2) in the main manuscript are written as:

$$\begin{pmatrix} S_{xx} \\ S_{yy} \\ S_{zz} \\ S_{yz} \\ S_{xz} \\ S_{xy} \end{pmatrix} = \begin{pmatrix} s_{E11} s_{E12} s_{E13} s_{E14} s_{E15} s_{E16} \\ s_{E21} s_{E22} s_{E23} s_{E24} s_{E25} s_{E26} \\ s_{E31} s_{E32} s_{E33} s_{E34} s_{E35} s_{E36} \\ s_{E41} s_{E42} s_{E43} s_{E44} s_{E45} s_{E46} \\ s_{E51} s_{E52} s_{E53} s_{E54} s_{E55} s_{E56} \\ s_{E61} s_{E62} s_{E63} s_{E64} s_{E65} s_{E66} \end{pmatrix} \begin{pmatrix} T_{xx} \\ T_{yy} \\ T_{zz} \\ T_{yz} \\ T_{xz} \\ T_{xy} \end{pmatrix} + \begin{pmatrix} d_{11} d_{21} d_{31} \\ d_{12} d_{22} d_{32} \\ d_{13} d_{23} d_{33} \\ d_{14} d_{24} d_{34} \\ d_{15} d_{25} d_{35} \\ d_{16} d_{26} d_{36} \end{pmatrix} \begin{pmatrix} E_x \\ E_y \\ E_z \end{pmatrix}$$

$$\begin{pmatrix} D_x \\ D_y \\ D_z \end{pmatrix} = \begin{pmatrix} d_{11} d_{12} d_{13} d_{14} d_{15} d_{16} \\ d_{21} d_{22} d_{23} d_{24} d_{25} d_{26} \\ d_{31} d_{32} d_{33} d_{34} d_{35} d_{36} \end{pmatrix} \begin{pmatrix} T_{xx} \\ T_{yy} \\ T_{zz} \\ T_{yz} \\ T_{xz} \\ T_{xy} \end{pmatrix} + \varepsilon_0 \begin{pmatrix} \varepsilon_{rT11} \varepsilon_{rT12} \varepsilon_{rT13} \\ \varepsilon_{rT21} \varepsilon_{rT22} \varepsilon_{rT23} \\ \varepsilon_{rT31} \varepsilon_{rT32} \varepsilon_{rT33} \end{pmatrix} \begin{pmatrix} E_x \\ E_y \\ E_z \end{pmatrix}$$

The Voigt notation helps to represent symmetric tensors by reducing their order. The Voigt form of compliance, coupling, and relative permittivity matrices are illustrated below in **Table S1**.

Compliance coefficient	Coupling coefficient	Relative Permittivity coefficient
$s_{E11} = s_{E22}$<br>$s_{E13} = s_{E31} = s_{E23} = s_{E32}$<br>$s_{E12} = s_{E21}$<br>$s_{E44} = s_{E55}$<br>$s_{E66} = 2(s_{E11} - s_{E12})$	$d_{ET31} = d_{ET32}$<br>$d_{ET15} = d_{ET24}$	$\varepsilon_{rT11} = \varepsilon_{rT22}$<br>$\varepsilon_{rT33}$

$$s_E = \begin{pmatrix} s_{E11} & s_{E12} & s_{E13} & 0 & 0 & 0 \\ s_{E21} & s_{E22} & s_{E23} & 0 & 0 & 0 \\ s_{E31} & s_{E32} & s_{E33} & 0 & 0 & 0 \\ 0 & 0 & 0 & s_{E44} & 0 & 0 \\ 0 & 0 & 0 & 0 & s_{E55} & 0 \\ 0 & 0 & 0 & 0 & 0 & s_{E66} \end{pmatrix}$$

$$d_{ET} = \begin{pmatrix} 0 & 0 & 0 & 0 & d_{ET15} & 0 \\ 0 & 0 & 0 & d_{ET24} & 0 & 0 \\ d_{ET31} & d_{ET32} & d_{ET33} & 0 & 0 & 0 \end{pmatrix}, \qquad \varepsilon_{rT} = \begin{pmatrix} \varepsilon_{rT11} & 0 & 0 \\ 0 & \varepsilon_{rT22} & 0 \\ 0 & 0 & \varepsilon_{rT33} \end{pmatrix}$$

Table S1. The Voigt form of compliance (s_E), coupling (d_{ET}), and relative permittivity (ε_{rT}) matrices.

The material parameters used in our simulation are summarized below in **Table S2**. In addition to a basic set of parameters such as density (ρ, in kg/m^3), Young's modulus (E, in Pa), and Poisson's ratio (ν, dimensionless) (see **Table S2(a)**), the use of an electrostrictive material requires us to enter the elements of the above matrices (s_E, d_{ET} and ε_{rT}) (see **Table S2(b)**). It is noted that since our simulation adopts an experimentally benchmarked model, the d_{33} parameter takes a range of values rather than a single, fixed value; depending on the thickness of the BTO layer, d_{33} changes from 3.13×10^{-13} (C/N) to 7.38×10^{-12} (C/N). These values are based on experimental measurements with the BTO thin films [2], which are quite different from the built-in value in the COMSOL material library (8.56×10^{-11} C/N). For values that are not directly available from the experimental work, we used the extrapolation technique by implementing the line graph equation and using the graph reading software [3].

Materials	Parameters		
	ρ	E	ν
Tungsten (W)	19,350	411×10^9	0.28
Barium Titanate (BTO)	6,020	128×10^9 [4]	0.35 [4]
Strontium Titanate (STO)	5,110 [5]	272×10^9 [6]	0.232 [7,8]
Silicon (Si)	2,329	202×10^9	0.27

(a)

Barium Titanate (BTO)		
s_E	s_{11}	8.05×10^{-12}
	s_{12}	-2.35×10^{-12}
	s_{13}	-5.24×10^{-12}
	s_{33}	1.57×10^{-11}
	s_{44}	1.84×10^{-11}
	s_{66}	8.84×10^{-12}
d_{ET}	d_{15}	3.92×10^{-10}
	d_{31}	-3.45×10^{-11}
	d_{33}	$3.13 \times 10^{-13} \sim 7.38 \times 10^{-12}$ [2]
ε_{rT}	ε_{11}	2,920
	ε_{33}	168

(b)

Table S2. The summary of key material parameters used in the simulation. (a) shows density (ρ, in kg/m^3), Young's modulus (E, in N/m^2), and Poisson's ratio (ν, the dimensionless ratio of lateral to longitudinal strains) for all materials used while (b) lists the parameters specifically required for the piezoelectric material (BTO), including the compliance (s_E), coupling (d_{ET}), and relative permittivity (ε_{rT}) matrix elements. The strength of the out-of-plane tensile strain in the BTO layer, represented by the d_{33} value in the table, plays a major role in determining the stress induced in the neighboring channel (STO) layer. The incorporation of experimental values (especially important due to the dependence on the film thickness) of d_{33} for BTO thin films [2] ensures the precise emulation of experimental situations.

Regarding other simulation details, it is important to note that tetrahedral extra-fine meshing was applied only to the STO layer for computational efficiency. Because the focus of our simulative work is to investigate the maximum stress level induced at the STO layer, normal mesh conditions applied to the other layers (gate electrode, piezoelectric layer, and substrate) did not alter the main scope of our work or the simulation result.

C-AFM Experiments

In order to perform the electromechanical coupling experiments (i.e., stress-induced conductivity modulation) with the (Bruker Innova Series) C-AFM instrument, the control parameter of the experimental setup ($V_{setpoint}$) needs to be precisely converted to the mechanical pressure (N/m^2). This conversion is based on the Hooke's law (i.e., Force is the product of spring constant and displacement). With the spring constant (N/m) and the deflection sensitivity (m/V) values separately obtained from the built-in calibration software, the amount of applied forces could be calculated for a given value of the setpoint voltage (displacement is the product of deflection sensitivity and setpoint voltage). The pressure is then calculated by dividing the force by the contact area where the force is applied. For each pressure applied, the resistance was measured using the conductive tip (Asyelec.01-R2), and the conductivity of the STO thin film was finally calculated. All relevant equations are summarized below.

Equations

$$\Delta x = V_s * y$$

$$F = k * \Delta x$$

$$P = F/A$$

$$\rho = (A*R)/t$$

$$\sigma = 1/\rho$$

Δx: displacement, **V_s**: Setpoint voltage
y: deflection sensitivity
F: force, **k**: spring constant
P: Pressure, **F**: force, **A**: area
ρ: Resistivity, **R**: resistance,
t: thickness of the sample, **σ**: conductivity

The spring constant calibration was performed in the contact AFM mode, where a thermal tune spectrum was plotted to fit with a simple harmonic oscillator model with the resonance frequency and Q-factor parameters. The deflection sensitivity was calculated in the C-AFM mode, performing the point spectroscopy (photodetector signal vs. piezo-movement) measurements on the sample and extracting the slope of the repulsive portion in the resulting plot.

Memristive Switching in STO

Fig. S2 shows the measured current-voltage (I-V) characteristic of the STO-based two-terminal device, fabricated using the simple shadow mask technique (top electrode contact/STO/silicon/bottom electrode contact). A clear hysteresis is observed in the DC bias sweep, indicating that oxygen-vacancy controlled memristive switching can enable ON/OFF switching required for electrostrictive FET operations. In this demonstration, a relatively thin (9 nm-thick) film of single-crystalline STO was prepared by the MBE technique with a decent amount of oxygen vacancies introduced during the growth.

(see the next page for **Fig. S2**)

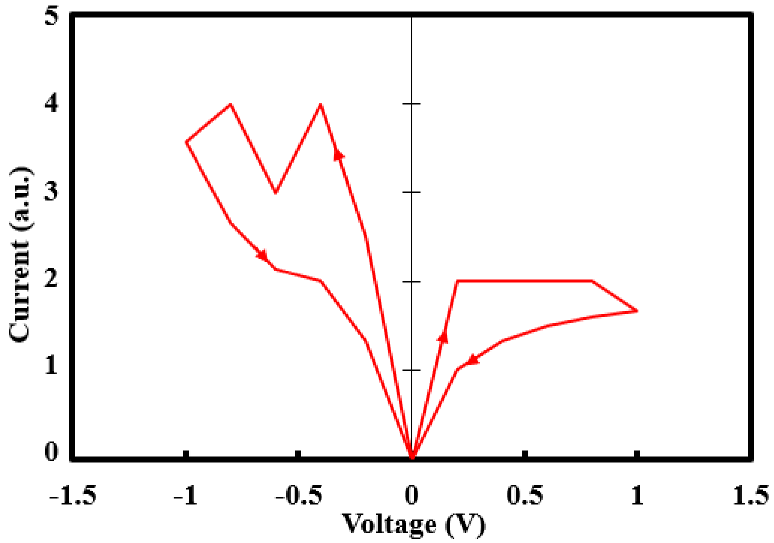

Figure S2. The memristive switching (I-V) curve of the STO thin film. Reversible switching between higher and lower conductance levels indicate that this material could be used as the FET channel.

References

1. Piezoelectric Materials: Understanding the Standards. *COMSOL Multiphysics* https://www.comsol.com/blogs/piezoelectric-materials-understanding-standards/ (2014).
2. Kelley, K. P. et al. Thickness and strain dependence of piezoelectric coefficient in BaTiO3 thin films. Phys. Rev. Materials 4, 024407 (2020).
3. graphreader.com - Online tool for reading graph image values and save as CSV / JSON. http://www.graphreader.com/.
4. Dent, A. C., Bowen, C. R., Stevens, R., Cain, M. G. & Stewart, M. Effective elastic properties for unpoled barium titanate. *Journal of the European Ceramic Society* **27**, 3739–3743 (2007).
5. Persson, K. *Materials Data on SrTiO3 (SG:140) by Materials Project*. https://www.osti.gov/dataexplorer/biblio/dataset/1208312 (2014) doi:10.17188/1208312.
6. Venkatesan, S., Kooi, B. J., De Hosson, J. T. M., Vlooswijk, A. H. G. & Noheda, B. Substrate influence on the shape of domains in epitaxial PbTiO 3 thin films. *Journal of Applied Physics* **102**, 104105 (2007).
7. Tse, Y. Y., Koutsonas, Y., Jackson, T. J., Passerieux, G. & Jones, I. P. Microstructure of homoepitaxial strontium titanate films grown by pulsed laser deposition. *Thin Solid Films* **515**, 1788–1795 (2006).
8. Taylor, T. R. et al. Influence of stoichiometry on the dielectric properties of sputtered strontium titanate thin films. *Journal of applied physics* **94**, 3390–3396 (2003).
